# Health Enhancing Physical Activity Policies in Poland: Findings from the HEPA PAT Survey

**DOI:** 10.3390/ijerph19127284

**Published:** 2022-06-14

**Authors:** Aleksandra Romanowska, Agnieszka Morawiak, Catherine Woods, Liam Kelly, Kevin Volf, Peter Gelius, Sven Messing, Sarah Forberger, Jeroen Lakerveld, Nicolette R. Den Braver, Enrique García Bengoechea, Joanna Żukowska

**Affiliations:** 1 Faculty of Civil and Environmental Engineering, Gdansk University of Technology, 80-233 Gdansk, Poland; agnieszkamorawiak@wp.pl (A.M.); joanna@pg.edu.pl (J.Ż.); 2 Physical Activity for Health Research Cluster, Department of Physical Education and Sport Sciences, Health Research Institute, University of Limerick, V94 T9PX Limerick, Ireland; catherine.woods@ul.ie (C.W.); liam.kelly@ul.ie (L.K.); kevin.volf@ul.ie (K.V.); enrique.garcia@ul.ie (E.G.B.); 3 Department of Sport Science and Sport, Friedrich-Alexander-Universität Erlangen-Nürnberg, 91058 Erlangen, Germany; peter.gelius@fau.de (P.G.); sven.messing@fau.de (S.M.); 4 Leibniz Institute for Prevention Research and Epidemiology BIPS, 28359 Bremen, Germany; forberger@leibniz-bips.de; 5 Department of Epidemiology and Data Science, Amsterdam Public Health Research Institute, Amsterdam UMC, VU University Amsterdam, 1081 HV Amsterdam, The Netherlands; j.lakerveld@amsterdamumc.nl (J.L.); n.denbraver@amsterdamumc.nl (N.R.D.B.); 6 Research & Innovation Unit, Sport Ireland, D15 PNON Dublin, Ireland

**Keywords:** physical activity, Poland, HEPA PAT, public health, public policy

## Abstract

Insufficient physical activity (PA) is one of major risk factors for serious diseases and premature mortality worldwide. Public policies to enhance PA across society are recognized as an effective tool against the problem. This paper presents the results of a comprehensive assessment of national-level PA policy approach in Poland. A standardized survey of Word Health Organization named the Health-Enhancing Physical Activity Policy Audit Tool (HEPA PAT) was used for data collection. Content analysis and strengths, weaknesses, opportunities, and threats analysis (SWOT) were used to characterize various PA policy aspects, to appraise the current situation, and accommodate organizational and environmental factors that it is influenced by. The results show that the national PA policy approach has been constantly developing in Poland, but there is room for improvement in a number of areas. The most important weaknesses are the lack of clear leadership, no mechanisms in place to coordinate efforts undertaken at different levels, and lack of collaboration across different levels of government and across different sectors of economy. Providing an umbrella covering all PA promotion policies and activities is, therefore, a key issue to be addressed. The country should seize the opportunity coming from an increasing awareness of a healthy lifestyle among Polish society.

## 1. Introduction

Non-communicable diseases are responsible for almost three quarters of global deaths [1], and a lack of physical activity (PA) is one of their major risk factors [2,3]. Lee et al. [4] estimated that 6% of the burden of coronary heart diseases, 7% of type 2 diabetes, 10% of breast cancers, and 10% of colon cancers are caused by an inactive lifestyle. He also estimated that with a 25% increase in global PA level, more than 1.3 million deaths could be averted each year. Therefore, regular PA is an effective prevention strategy against numerous chronic diseases and may reduce their risk by 20–30% [5]. Despite the empirical evidence on the health benefits of PA [4,6], over a quarter of the world’s adult population and more than three quarters of the world’s adolescents do not adhere to current PA guidelines [7]. In addition, no improvement has been noticed in global PA levels since 2001 [7]. For this reason, promotion of PA is a topic of great importance for both the World Health Organization (WHO) and the European Union (EU) [8,9].

Since 1995, the WHO has been providing evidence-based recommendations for PA [10]. The newest were published in 2020 [11]. The document provides details on the amount, type, and frequency of PA that is needed for health and well-being for specific population groups, such as small children, school-aged children, adults, the elderly, or pregnant women. According to Oja and Titze [10], PA recommendations targeting individuals are insufficient to achieve desired PA levels across the population, however, they may provide foundations for actions to improve the situation and metrics for their monitoring. Thus, policies to promote PA may be used as a response to the global physical inactivity problem. Compared to individual-level interventions, policies are targeted at a broader audience, community, or population, and therefore, have a wider spectrum of influence [12]. Sallis et al. [13] define PA-related policies as ‘legislative, regulatory, or policy-making actions that have the potential to affect physical activity’. They may provide opportunities for increasing PA levels, provide funding for PA promotion, regulate the amount of obligatory sports at school, and coordinate activities undertaken at national or regional level [12,14]. Many studies provide evidence for the effectiveness of policies to promote PA [15,16,17,18]. Such policies have been successfully implemented in the areas of education, health, sport, urban design, or transportation. Taking the latter as an example, Brockman and Fox [19] assessed the impact of transport-related policies introduced in Bristol in 1997–2007 that included increasing parking fees, subsidies for bicycle purchases, introducing a car-sharing system, and public transport discounts, concluding that walking to work increased by 11%. Another policy introduced in Cambridge, which focused on increasing access to walking/cycling routes and places for PA, has significantly increased children’s PA levels [20].

According to the WHO [21], the central role in promoting healthy lifestyles and creating an environment that encourage behavior changes belongs to the governments. The role is not limited to the health sector but also to other sectors such as transport, education, sports, or the environment. National governments have a steering and coordinating role in implementing strategies and meeting recommendations developed by WHO [21,22] or EU [8] at the national level. They have resources to provide effective legislations, develop programs, and ensure an appropriate infrastructure, funding, monitoring, or research opportunities. National policy is an important platform for governments to develop, coordinate and deliver large scale actions across multiple sectors, involve stakeholders, assign roles and responsibilities, define common objectives and gain visibility at the political level [23]. However, not all countries use the potential their governments have in PA promotion.

To assess the national PA policy potential and approaches, the WHO proposed a policy audit tool (Health-Enhancing Physical Activity Policy Audit Tool—HEPA PAT) and a wide variety of countries have used it so far [24,25,26,27]. The HEPA PAT is a standardized questionnaire designed to collect comprehensive, systematic, and comparable data on the approaches to PA promotion at the national policy level [23,28]. The completed HEPA PAT questionnaire provides a comprehensive overview on the current status of a country’s approach to enhancing PA, allows to identify strengths and weaknesses of the current national policy approach, indicates synergies and discrepancies between national level policy documents, and identifies whether there is enough communication and collaboration between sectors [23]. Finally, it allows for country comparisons [23,24], communication of good practices, and success stories to be used by other countries.

This paper presents the results of a comprehensive assessment of national-level PA policy approach in Poland based on the data systematically collected with the use of HEPA PAT questionnaire. The paper aims to answer the following questions: What are the strengths and weaknesses of the Polish PA policy approach? What policy areas need to be improved? The findings from this study will contribute to advanced knowledge in this particular area and to help policymakers and practitioners design and plan actions to increase PA levels among the whole of society. The rationale for this study is aligned with the work of the Policy Evaluation Network (PEN; https://www.jpi-pen.eu/). PEN aims to develop a consolidated approach to policy evaluation across Europe by developing and prioritizing an agreed set of indicators, measured using harmonized instruments that ideally can be used by existing monitoring and surveillance systems [29]. This study was also the first step in developing the prototype of ‘Physical Activity Environment Policy Index’, a tool that can be used to independently monitor and benchmark public sector PA policies and actions.

## 2. Materials and Methods

To identify and assess the current state of the national-level PA policy approach in Poland, the WHO’s audit tool HEPA PAT version 2.0 was used [28]. The questionnaire consists of 29 questions in 10 subject areas (Table 1). The process of completing the HEPA PAT was supervised by the project coordinator (JŻ) and involved national stakeholders from several sectors such as sports, health, education, and transport.

The process of data collection started in March 2019 and was completed in July 2019. It consisted of the following stages: (i) identification of stakeholders; (ii) collecting the data from the 2018 survey for the EU Monitoring Framework after obtaining consent from the national PA Focal Point and the WHO; (iii) desk research conducted by the authors, including the identification and the review of existing policy documents, programs, activities carried out at the national level and related to PA promotion; (iv) in-depth expert interviews to obtain additional information and input for the HEPA PAT; (v) completing the HEPA PAT questionnaire by the authors. The in-depth interviews with experts involved the representatives of the Polish Ministry of Health, the Ministry of Sport and Tourism, the Ministry of Infrastructure, and the Ministry of Education (currently the Ministry of Education and Science). The participants were selected based on their expertise in the PA policy setting and policy implementation.

Similarly, data were collected in Ireland, The Netherlands, and Germany [23,24,27]. While the results of this four-country comparison have been published elsewhere, this article presents the results for Poland in a higher level of detail.

In order to characterize the various policy aspects related to the development, implementation, monitoring, and evaluation of PA policies, a content analysis of the completed HEPA PAT questionnaire was performed. This is a research technique widely used for making inferences from analyzing documents that were generated or obtained in the course of research [30]. The HEPA PAT categories served as basic themes for the analysis. The research questions were raised to understand the information of how it is presented: How is the process (e.g., implementation, evaluation) organized? What are the outcomes? What lacks in the process? The conclusions were drawn from the HEPA PAT content to the context, which is the history and current state of PA policy approach in Poland, using the experience and knowledge by researchers directly involved in the data collection process.

To summarize the results, a SWOT (strengths, weaknesses, opportunities, threats) analysis was used. This is a powerful tool widely used in strategic planning and management, which is helpful to appraise the state of the art and accommodate organizational and environmental factors that influence the current situation [31]. SWOT analyses have been adopted in some qualitative studies to evaluate policy approaches in recent years, including energy planning strategies [32,33], the compressed natural gas industry, [34] or urban transport system [35]. It has also been applied to evaluate PA promotion in a household group survey [36]. In this paper, SWOT is used to structure the factors influencing and shaping PA promotion policy at the national level in Poland. For this purpose, the particular components of SWOT representing internal (strengths and weaknesses) and external (opportunities and threats) factors, respectively, were summarized in a 2 × 2 matrix. The matrix components were further divided into rows comprising factors in accordance with the adopted themes and matching them horizontally (i.e., strengths with corresponding weaknesses; opportunities with corresponding threats).

## 3. Results

### 3.1. Leadership and Partnership

Poland is a unitary state with a strong central government. In line with the EU recommendations on promoting health-enhancing PA (HEPA) across sectors [8], Poland has appointed a National PA Focal Point at the Ministry of Sport and Tourism, Department of Sport. The Ministry is responsible for the development and implementation of national sport and tourism strategies. It undertakes many activities to promote PA, but these are limited mainly to the area of sport. The Ministry does not provide any leadership and any umbrella for PA promotion activities implemented in other areas (i.e., health, transportation, and social policy).

Apart from the Ministry of Sport and Tourism, other government ministries have their own role in PA promotion. Table 2 presents information about ministries, their responsibilities, and how they are related to PA promotion. The Ministry of Health, responsible for health policy, develops and implements the national health strategy, which covers PA-related tasks. The Ministry of Family and Social Policy, responsible for the welfare of Polish families and the whole society, supports the development of care services and cares for the PA of seniors. The Ministry of Education and Science, responsible for teaching and education, supports the development of educational programs, including physical education (PE) classes at schools. Although the Ministry of Infrastructure, the Ministry of Investment and Development, and the Ministry of the Environment do not have special roles in PA promotion, they indirectly support PA by different programs devoted to active transport, active mobility, and development of green transport.

There are currently no special mechanisms or agencies to ensure the co-operation between particular ministries in the implementation of the PA policies at the national level. There is also no agency at the national level to promote and coordinate PA activities with the subnational level, either horizontally or vertically.

### 3.2. Policy Documents

The most important policy documents that influenced the shaping of the PA policy agenda in Poland were the National Health Programme 2007–2015 [37], the Sport Development Strategy until 2015 [38], and the Directions of Tourism Development until 2015 [39]. These programs covered the sectors of sport, health, education, and tourism. All were continued and covered the period until 2020 [39,40,41]. In addition to the mentioned programs, there are policy documents in other sectors that have implications for PA behavior; these are the Transport Development Strategy until 2020 [42] which appeared in transport sector, and the National Urban Policy 2023 [43], which is rooted in the urban planning sector. Key policy documents related to PA promotion are listed in Table 3.

While there are a number of current key policy documents expressing the intention to increase the national level of PA (e.g., [39,40,41,44]), there are no clear references across these documents and links to other documents. The only existing links refer to policies at the European level. An example is the Sport Development Programme 2020 [41], which takes the European Commission’s White Paper on Sport [45] as a basic document setting strategic guidelines for the role of sport in Europe and Poland.

### 3.3. Policy Scope and Implementation

Nearly all population groups, including preschool children, adolescents, individuals with disabilities, clinical populations, families, and migrant populations, are covered in the documents identified.

Although there is no nationwide mass media communication strategy to promote PA in Poland, some agencies are funding initiatives that utilize mass media, i.e., social media channels. However, no coordination of these initiatives is ensured.

On the other hand, there are many successful programs and interventions in Poland promoting PA in sectors such as health, sport, education, transport, and environment. The most successful and widespread programs in recent years were the National Talent Base, the Local Sports Animator, the Orlik 2012, and the Stop Abstention from PE Classes (Table 4).

### 3.4. PA Recommendations, Goals and Targets

Poland has official guidelines for recommended levels of PA in different age groups, including children of all ages, adults, and seniors [46]. In case of adults, the recommendations are also targeted to pregnant women and adults with chronic diseases, but not to people with disabilities. The document is based on the WHO recommendations and considers the results of the study on PA among Poles conducted in the years 2015–2017. The recommendations set minimum and optimal time of weekly PA recommended in particular groups and propose the type and intensity of activity that should be undertaken, i.e., for seniors, it is recommended to undertake 150 min of moderate PA per week, including exercises to improve balance, coordination, and to strengthen all body muscles.

Sedentary behavior is not addressed by any national recommendations. This is an important weakness of the Polish PA promotion system, while many countries provide recommendations in this regard (i.e., Estonia, Greece, and France) [47].

Improving the PA levels in Poland was the main goal of the Sport Development Programme 2020 [41]. According to the program, the percentage of residents that undertake the recommended amount of PA was to be increased by 3.5% and the percentage of residents that never do exercise or play sport was to be lined up with the EU average. Mid-term evaluation indicates that even though the situation has improved, Poland is still far away from achieving these goals [48].

### 3.5. Surveillance

Poland has been conducting regular PA monitoring among different age groups for the past ten years. In 2016, the country joined the WHO’s European Childhood Obesity Surveillance Initiative (COSI) [49]. Within the initiative and financed by the National Health Programme [40], a standardized survey and body measurements were conducted among 8-year-olds in 2016 and 2018. The results of the study provide information on the level of PA and sedentary behavior of school pupils. According to the latest results, a large proportion (61%) of children do 1–2 h of PA daily, however there is also a significant proportion (10–20% depending on the type of day) who are not active at all. The time spent on watching TV or using electronic media is approximately 1.5 h on an average school day and 2.5 h during non-school days.

Since 2010, Poland has been carrying out the regular monitoring of PA among children aged 11–15 using the HBSC methodology [50]. The studies were conducted in 2010, 2014, and 2018. The latest study [51] showed that the recommended PA level in Poland is achieved only by 17.2% of teenagers. Most of the teenagers (approx. 60% during working days and 80% during weekend) spend more than two hours a day sitting in front of TV, computer, tablet, or smartphone, and this percentage is increasing.

Pilot studies are also carried out in other age groups, e.g., among older youth (17–19) and in pre-school children. The health and PA level among Polish residents have also been a subject of a study based on the European Health Interview Survey (EHIS) [52] in 2009, 2014, and 2019. Since 2014, the Ministry of Sport and Tourism has been committing research on the PA levels of Polish residents on an annual basis, using the International Physical Activity Questionnaire (IPAQ) [53]. The aim of the study is to indicate the percentage of Polish residents meeting the WHO’s recommendations regarding the time spent on PA (according to the latest results these are met by only 21.8% of residents aged 15–69 [54]).

A regular monitoring of PA is also provided by the Main Statistical Office (2008, 2012, and 2016), using a questionnaire survey in households. The respondents are asked about their perceived physical fitness and the level and regularity of PA.

Despite the amount of data collected and updated in the above-mentioned studies, most policy documents do not take the results into account. The exception is the National Sport Development Programme 2020 [41], whose goals were based on the diagnosis using surveillance data, as well as the data used for program monitoring. The existing data are also not commonly used for PA promotion activities. However, some good practices can be found. In 2009–2012, the National Supreme Audit Office has investigated the organization of PE classes and the levels of participation. The results were alarming—physical fitness among children decreased, they often had bad posture, and they experienced the fastest weight increase in Europe. The PE classes were also neglected by schools and teachers; according to the results, 65% of the classes were not even conducted. In response, the Ministry of Sport and Tourism has initiated the promotional campaign Stop Abstention from PE classes (Table 4), which aimed to fight against the scourge of PE exemptions and increasing parents’ awareness about the importance of PA for children’s health and fitness.

### 3.6. Evaluation

Among the policies in place, only the National Sport Development Programme [41] was developed based on previous program evaluation results and includes a detailed evaluation plan. The evaluation is done at the strategic (achievement of program goals) and operational level (implementation of the program action plan). An evaluation report is published on an annual basis.

One-time evaluation was also done for the National Health Programme [55]. However, this evaluation was only based on the qualitative assessment of the organization of particular initiatives and their strengths and weaknesses, but did not assess whether the goals of the program were achieved.

### 3.7. Funding and Commitments

According to the Ministry of Finance, in 2015, public expenditure on sport and PA amounted to 1.1 billion euro, of which over 90% was spent by local governments. This means that local governments play a key role in financing sport and recreation from public sources. Local government units allocate much more funds to physical activity promotion than the state budget. The public expenditures on sport and PA represent 0.4% of the national GDP [56]. The budget spent per capita was 43 euro in 2015 and increased to 60 euro in 2019; however, it is still 50% lower than the EU average [57,58].

At the national level, the delivery of PA-related policy is funded from several sectors (public funds). The largest funding comes from the sport and recreation sector and the transport sector; much less is dedicated from the sectors of health, education, tourism and social affairs. A large amount (70 million euro) was dedicated for financing initiatives within the National Sport Development Programme [38].

### 3.8. Capacity Building through a National Network

National networks that support professionals in PA promotion primarily include publicly funded associations and informal networks. An example is the National Talent Base (https://www.narodowabazatalentow.pl), which collects and shares data and knowledge about the level of physical condition of children and adolescents as well as helps to discover young talents in sport. Another example is the Polish Active Mobility Association (PUMA) that supports local governments in promoting and improving conditions for active transport.

### 3.9. Summary

Table 5 summarizes the results of the Polish HEPA PAT obtained from both the expert interviews and the Polish HEPA Focal Point with the help of the SWOT analysis. It indicates the strengths and weaknesses of the PA policy approach and highlights external opportunities and threats that may influence its future development.

## 4. Discussion

HEPA PAT turned out to be a powerful tool for identifying the strengths and weaknesses of PA policy approach in Poland. The summary of results of its application presented in Table 5 allows conclusions to be drawn on what are the successes, what are the failures, where is a room for improvement, and what needs to be done from scratch regarding the efforts to increase the national PA level.

It is clear that the most important weakness of the Polish PA policy approach is the lack of clear leadership and the lack of mechanisms to coordinate efforts at different levels and to ensure effective collaboration (both vertically across different levels of government and horizontally across different sectors). In the meantime, governments are encouraged by the WHO to set up a national coordinating mechanism that addresses PA within the context of a comprehensive plan for noncommunicable-disease prevention and health promotion. Local authorities should be closely involved. WHO recommendations underlie that multisectoral and multidisciplinary expert advisory boards should be established. They should include technical experts and representatives of government agencies, and have an independent chair to ensure that scientific evidence is interpreted without any conflict of interest [21]. None of these currently exist in Poland.

There are government structures that are actively promoting PA in the areas they are responsible for (i.e., sports, health, urban policy, transport, etc.), but their potential as policy makers is untapped. Their actions are undertaken independently and without linkage to policies in other sectors, even when they have the same goal of making the Polish society healthier and more active.

Not only governmental structures, but also many processes, are already in place and dealing with PA promotion. Providing an umbrella covering all PA policies and activities is, therefore, an extremely important issue to be addressed in Poland. This is exactly what the WHO [21] recommended to its member states, emphasizing that it is governments that create the conditions for change in promotion of healthy lifestyles.

There are also strengths of the Polish PA policy approach. One of these is a regular PA monitoring. Data are collected in different population groups (i.e., adults, school-aged children, and adolescents) and mostly on a regular basis, with the use of different methodologies. However, the existing monitoring tools and methods do not account for different external and internal conditions that may be influential (e.g., age group and weather conditions). The surveillance data come mainly from self-reported surveys (e.g., IPAQ). According to Winckers et al. [59], who compared IPAQ results with accelerometer data, using subjective PA measures leads to a general overestimation of PA; low data validity is especially observed in a lower-educated adult group. This may raise a question of validity of the surveillance data, which is not complemented with objective measurements.

Despite the amount of data being collected, these are rarely used in policy formulation or evaluation. The only policy document that is based upon the data and uses these data for evaluation is the National Sport Development Program [41], developed by the Ministry of Sport and Tourism. Lack of a clear evaluation plan makes it impossible to monitor whether other programs or interventions have met their objectives and how they should be changed in the future to be more effective. With single exemptions, the data are also not used for PA promotion or communicated to the society.

Based on WHO guidelines [11], Poland sets the recommended daily PA for most population groups. However, the recommendations are communicated to the society to a low extent. It is not evaluated to what extent people are aware of their existence. An important problem identified during the HEPA PAT data collection process was the lack of Polish recommendations on reducing sedentary behavior, which affects a large share of population [57].

While the surveillance data enable regular monitoring and evaluation, not all major policy documents have an evaluation plan included. The need for improvement in policy evaluation both in Poland and in other countries was also highlighted by Gelius et al. [24].

The results of this study show the potential of the efforts taken so far in improving PA level across society. Numerous actions are undertaken at different levels; different policy documents are in place, different sectors are engaged, and a large amount of data are collected. What is missing is effective coordination making the linkage between different activities and bodies engaged, and between actions undertaken at different levels (i.e., national, regional, and local). Poland may benefit from this comprehensive assessment in numerous ways. The country may use the results to prioritize actions regarding PA promotion that has to be undertaken and formulate recommendations for further activities. The methodology may be used by the country in order to monitor particular areas of PA policy approach. Other countries may also benefit from this comprehensive assessment. They may use the adopted methodology in order to identify strengths and weaknesses of their PA promotion policy approach; they may use the results to identify and compare the weak and strong points of the PA policy. Finally, they may use some practices that were assessed as successful in Poland.

The present study suffers from some limitations. The research was conducted at the national level only, and no regional or local level PA-related activities were included. Thus, the subnational level requires investigation and will be a subject of further research.

As data collection took place in 2019, the influence of the COVID-19 pandemic on policies promoting physical activity in Poland could not be assessed in this study.

## 5. Conclusions

The study provides a comprehensive overview and assessment of the efforts undertaken to promote PA at the national level in Poland. The results confirm that the national PA policy approach has been constantly developing in Poland and currently includes multiple sectors and covers major population groups, although there is still room for improvement in most fields. While the need for improvement is recognized in most countries [24,60], lack of clear leadership and coordination of policies in Poland is almost an impassable barrier in increasing effectiveness of PA promotion in Poland. The findings from this study may help policymakers and practitioners design and plan actions to increase PA levels among the whole of society.

## Figures and Tables

**Table 1:** The overview of the HEPA PAT structure.

Section	Goals	Data Collection		
		Focal Point	Desk Review	Expert Survey
Background information and country context	To give a brief overview of the country’s organization, government structure, distribution of responsibilities across sectors and ministries, and entities involved in PA promotion at the national level.		X	
Leadership and partnerships	To identify what entities provide leadership for PA promotion, whether there are mechanisms or agencies that ensure and coordinate cross-sectoral collaboration.	X		
Policy documents	To give an overview of how the PA promotion agenda was shaped in past policy documents; to indicate the current policy documents important for further shaping the PA promotion agenda across sectors; to assess the policy setting process; to show the link with other national policy documents and with documents at the global or EU level.	X	X	
Policy scope, content and implementation	To identify population groups targeted by PA promotion activities; to describe communication processes; to give examples or case studies of large-scale PA promotion activities across sectors.	X		X
Recommendations, goals and targets	To give an overview of national recommendation on physical activity and sedentary behavior and how different population groups are targeted; to identify national goals or targets set in national policy documents.		X	
Surveillance	To collect information on how PA and sedentary behavior are monitored, what data are being collected, and what is the frequency of data collection; to show whether and how the data are used in national PA promotion policies.	X	X	
Evaluation	To identify whether the past national policy documents were evaluated and how; what were the results and whether and how they were used in new policies.	X	X	
Funding and commitments	To give an overview of funding for PA-related policies and interventions; to identify what is the political commitment to the national PA promotion agenda.			X
Capacity-building through a national network	To identify whether there is any professional network or system to link and support professionals involved in PA promotion.			X
Experience of policy implementation, progress and remaining challenges	To indicate the main achievements and challenges related to country level PA promotion; to give experience-based recommendations for other countries.			X

**Table 2:** The role of Polish ministries in PA promotion.

Name of the Ministry	Main Competences	Competences Related to PA Promotion
Ministry of Sport and Tourism	Development of the general and professional sport	Commissions PA monitoring; implements actions to enhance PA among society and specific population groups; develops and implements the national sport strategy.
Ministry of Health	Health policy development and implementation	Implements actions to prevent faulty posture in children and adolescents; establishes rules on medical eligibility for PE classes; develops and implements the national health program, including actions to enhance PA level among society.
Ministry of Family and Social Policy	Responsible for the welfare of Polish society	Supports the development of care services; implements actions to increase awareness and enhance PA among seniors.
Ministry of Education and Science	Teaching and education	Supports the development of health education and PE educational programs in schools; supports actions towards preventive healthcare for school children.
Ministry of Infrastructure	Transport infrastructure development and ensuring its rational use	Indirectly involved in PA promotion by implementing programs devoted to green and active transport, including active transport in strategies to promote low-carbon and compact cities, including active transport in strategies to reduce emissions from transportation.
Ministry of Investment and Development	Construction, housing urban planning, and development	
Ministry of Environment	Environment protection	

**Table 3:** Key PA policy documents in Poland.

Name	Sector	Main Goal(s)
The National Health Programmes 2007–2015 and 2016–2020	Health	To improve population health and life quality; to reduce inequities in health.
The National Sport Development Strategy 2015 and Sport Development Programme 2020	Sport, Education	To achieve the optimal level of pro-health behavior among different groups of Polish society by promoting sport and enhancing physical activity.
The Directions of Tourism Development until 2015 and until 2020	Tourism	To strengthen the development of competitive, innovative, and sustainable tourism that favors the country’s socio-economic development and increases competitiveness of Polish regions.
The Transport Development Strategy until 2020	Transport, Infrastructure	To increase the accessibility, safety, and efficiency of transport through the development of a coherent, sustainable, and user-friendly transportation system.
The National Urban Policy 2023	Urban planning, Infrastructure	To strengthen the sustainable development of cities and urban areas; to improve life quality of residents.

**Table 4:** Selected PA-related programs and interventions implemented in Poland.

Name	Description
The National Talent Base	A publicly available program that monitors and tests the level of physical fitness of youth. (https://narodowabazatalentow.pl/)
The Local Sports Animator	A systematic project promoting PA and sports among children and youth by enabling them to participate in extracurricular and out-of-school sports activities (https://orlysportu.pl/).
Orlik 2012	A program which provided for the construction of modern sport facilities in every municipality in Poland.
Stop Abstention from PE Classes	A program aimed at promoting PA among school-aged children and adolescents and encouraging them to attend PE classes.

**Table 5:** SWOT analysis of PA promotion in Poland.

Strengths	Weaknesses
There is a WHO focal point designated in the Ministry of Sport and Tourism.	There is no leader agency (nor leadership) responsible for PA promotion.
There are ministries directly (sport) and indirectly (i.e., health, transportation, and social policy) involved in PA promotion.	There is no cross-sectoral coordination on the national level. There is no link between policies, programs, and interventions implemented by different bodies (e.g., ministries) and no coordination of their activities. There is no vertical coordination between national and local level PA promotion activities. There is no umbrella strategy at the national level (the implemented programs and interventions are not a part of any wider PA promotion strategy).
There is a PA promotion policy in place (the National Sport Development Programme). There are several policies that indirectly link to PA promotion (in the area of i.e., health or transportation). There are many successful programs and interventions to promote PA in sectors like health, sports, education, transport, and environment.	A clear PA promotion policy is limited to the area of sport. There is no central policy document that would serve as an umbrella for PA-related policies in the areas of sport, health, transportation, etc.
There is a regular monitoring of PA levels across different society groups, especially children and adolescents. There is a publicly available tool (the National Talent Base) to measure physical fitness among youth using systematic measurements.	The existing monitoring tools and methods do not account for different external and internal conditions (e.g., age of participants and weather conditions). There is no evaluation of implemented programs and interventions; new programs and interventions are usually not based on previous evaluation results.
National recommendations on increasing PA include a wide range of age and social groups; including pregnant women and elderly people.	No recommendations are targeted at people with disabilities. There are no national recommendations on reducing sedentary behavior among different population groups.
The existing policy documents strongly refer to the relevant European policies.	There are no clear cross-sectoral links between policy documents.
	There is no national communication strategy regarding PA promotion.
**Opportunities**	**Threats**
There is an increasing awareness of a healthy lifestyle among Polish society; it is trendy to be active and fit.	There is a social tolerance for inactivity. Sedentary behavior is progressing in society; there is an alarming and increasing trend in time spent in front of electronic devices, especially among children, adolescents, and young adults.
Availability of open sport infrastructure constantly increase (e.g., new outdoor gyms are financed by governmental program).	Financing of new PA infrastructure and opportunities strongly depends on the country’s economic situation which is not constant.
There are many bottom-up initiatives by local governments and NGOs that supports PA promotion (promotional campaigns, competitions, mass events, marathons, etc.).	Implementation of many PA promotion interventions is affected by and depends on COVID-19 conditions.
There are structures in Polish parliament responsible for PA promotion related tasks.	The activity of parliament’s structures designated to promote PA is very low and isolated.

## Data Availability

The dataset analyzed during the current study, which is the completed HEPA PAT questionnaire, is available in the Supplementary Materials.

## Supplementary Materials

The following supporting information can be downloaded at https://www.mdpi.com/article/10.3390/ijerph19127284/s1. Health-Enhancing Physical Activity (HEPA) Policy Audit Tool (PAT) Version 2 POLAND.
