# Supplementary material for: Health Enhancing Physical Activity Policies in Poland: Findings from the HEPA PAT Survey
Source: Int J Environ Res Public Health. 2022 Jun 14;19(12):7284. doi: 10.3390/ijerph19127284 (PMC9223836; doi:10.3390/ijerph19127284)
Supplement: Supplementary file 1 [file ijerph-19-07284-s001.zip › ijerph-1716920-supplementary.pdf]

Health-enhancing physical activity (HEPA) policy audit tool (PAT)

Version 2

POLAND

Draft number:
Date: November 2019

Completed by:

Joanna Zukowska	Gdansk University of Technology
Lech Michalski	Gdansk University of Technology
Anna Gobis	Gdansk University of Technology

Lead author: Joanna Zukowska

Contact details: +48 608054458 / joanna.zukowska@pg.edu.pl

Glossary

The following definitions are proposed in order to support the collection of relevant information for the PAT (based partly on Report of the global survey on the progress in national chronic diseases prevention and control (1)). However, it is not unusual for terms to be used interchangeably; for example, in one country a document may be called a policy, while in others a similar document could be labelled as action plan or strategy.

Action plan	An action plan should identify **who does what** (type of activities and people responsible for implementation), **when** (time frame), **how** (approaches, activities and interventions) and for **how much** (resources). It should ideally also have an inherent mechanism for monitoring and evaluation. An action plan can be part of a policy (see below) or be an independent document.
(Health-enhancing) physical activity (HEPA)	This is any form of physical activity that benefits health and functional capacity, without undue harm or risk.
Policy	A policy is a written document that contains priorities, defines goals and objectives, and is usually issued by (part of) the public administration. It can contain or be accompanied by an action plan (see above).
Programme	A programme is a set of measures or a single (but large-scale) long-term activity, which may or may not be related to a policy document. A programme can contain different types of activities, such as social marketing campaigns, promotional events, specific interventions or initiatives in different settings, and can be time limited or open ended.
Strategy	A strategy is defined by a long-term plan designed to achieve national goals (in this case, to promote health and prevent diseases).
Surveillance / monitoring system	A health surveillance/monitoring system is the continuous, systematic collection, analysis and interpretation of the health-related data needed for the planning, implementation, and evaluation of public health practice.
Sedentary behaviour	Sedentary behaviour is any waking behaviour involving very low energy expenditure and a sitting or reclining posture.

Disclaimer
The responses to the audit tool questions, as well as the conclusions and views resulting from the use of the tool, are the responsibility of its users and do not reflect the views of the WHO.
The questions and explanatory text in this unprotected version may not be changed in any way.

© World Health Organization 2015

SECTION 1
Background information and country context

1a. Please provide a brief overview of the **government structure** in your country (about 200-400 words). For example, briefly outline whether your country has a centralized or federal system and on which government level the main responsibility lies for issues such as health, sport, education, transport, environment and urban planning policy. *For examples relating to this and the other PAT questions, refer to the WHO website* (www.euro.who.int/hepapat).

The Republic of Poland is a parliamentary democracy under the 1997 Constitution. The Parliament is bicameral and is composed of an upper house, the Senate (*Senat*), and a lower house, the *Sejm*. None of the Chambers directly represent local and regional authorities. The Head of State is the President of the Republic and the Government is led by the Prime Minister.

Poland is a unitary State organised on a decentralised basis. In 1989, Municipalities were introduced. Then, two other levels of self-government – at the county and regional levels – were introduced.

National government provides for policy and the framework for sports and physical activity. However, the self-government level decide themselves to what extent (and if) they follow national policy and provide for and support sports infrastructure, programs, activities etc.

Health
Local and regional level bodies are responsible for performing the tasks (youth health, health care for the elderly, etc.) that are described in the public health act. National priorities provide a framework.

Sports
In Poland the topic of sports is covered by the Ministry of Sport and Tourism. Sports accommodations are under the responsibility of all administration levels: national, regional and local level. Only 2% of sports expenses come from ministerial budget. The rest is financed by self-government.

Education
In Poland there is public education available in the form of municipal schools. The government finances the public education. Self-government is responsible for the management of a school (within the framework of national legislation) and the physical facilities. The education content remains at the discretion of the government.

Transport
The central government is responsible for decisions regarding the motorways, the provinces for provincial roads and municipalities for (even the smallest) local roads. Ensuring public transport, cycle paths as well as appropriate local parking is the responsibility of municipalities.

Environment
The Polish governemnt contributes to and supports the doctrine of sustainable development, stimulating green innovation and investment and financing activities aimed at the protection of the environment. At the level of municipalities, the system of building permits e.g. is linked to a system of environmental permits. Municipalities are responsible for the local regulations that are based on the national Environmental Act.

Urban planning
The urban landscape of Poland is characterised by its policentricity, with 913 cities in total. There are 17 Metropolitan European Growth Area, all over 200,000 inhabitants, among which the biggest are: Warsaw, Krakow, Gdańsk, Wrocław, Poznań, Katowice with the Upper-Silesian conurbation, Łódź and Szczecin. The whole urban population represents over 61% of the country population.

1b. Please briefly describe the governance at sub-national level (about 200-400 words) (e.g. at regional/provincial/cantonal/municipality level).
Provinces<br>Poland is currently divided into 16 provinces known as voivodeships. Administrative authority at voivodeship level is shared between a central government-appointed governor, called the voivode (usually a political appointee), an elected assembly called the sejmik, and an executive board chosen by that assembly. The leader of that executive is called the marshal.<br><br>*Counties*<br>Each voivodeship is divided into a number of smaller entities known as powiats (counties). The number of powiats per voivodeship ranges from 12 to 42. This includes both powiats proper (known as land counties), and cities with powiat status (city counties). Land counties have an elected council, which elects an executive board headed by the starosta. In city counties the functions of these institutions are performed by the city's own council and directly elected mayor.<br><br>*Municipalities*<br>The third level of administrative division is the gmina (also called commune or municipality). A powiat is typically divided into a number of gminas (between 3 and 19), although the city counties constitute single gminas. A gmina may be classed as urban, urban-rural, or rural. A gmina has an elected council as well as a directly elected mayor.
1c. Please provide a list of the **main government ministries** (e.g. health, sport, education, transport, environment and urban planning) in your national government that have a role in the promotion of HEPA (see Glossary for definition).<br>Please also include a brief description of the role(s) of these key HEPA-related government departments.<br>**Please note:** This question and Question 1d refer to the <u>national level</u>; please include information on the subnational level <u>only where relevant</u>, e.g. for countries with a strongly decentralized, federal system.<br>Example: list the ministries and their role(s).<br>The Ministry of Health, Welfare and Sport is responsible for sport, physical activity and health policy, initiation and delivery of frameworks for action plans/programmes, guidelines and subsidies. In addition, creating and maintaining links with other ministries and sectors concerning physical activity promotion is one of their tasks.
Ministry of Health is responsible for health policy in particular for development and implementation of national health strategies (which cover also the HEPA-related tasks).<br><br>Ministry of Sport and Tourism is a national focal point for HEPA in Poland. It is responsible for sport and PA policy and in particular for development and implementation of national sport and tourism strategies.<br><br>Ministry of Family, Labour and Social Policy is responsible for the welfare of Polish families and whole society. Regardin the HEPA it strongly supports the elderly people, who are growing in our country from year to year. Through the programs they support the development of care services and the activity of seniors.<br><br>Ministry of Education is responsible for teaching and educational programs (also those referring to PA Classes) at pre-schools as well as primary and secondary schools in Poland.<br><br>Ministry of Infrastructure, Ministry of Investment and Development and Ministry of the Environment do not have any major role in promotion of HEPA in Poland. Indirectly they support PA by programs devoted to active transport, active mobility and development of green transport.

1d. Please list any **other important national organizations**, outside of government, which are **actively engaged** in HEPA promotion. This could include national sporting organizations, NGOs, charities, advocacy groups, the academic or scientific community, among others.<br>Please provide a brief description of the role of these organizations (about 50–100 words).<br>Please add/remove rows as needed.

Society for the Promotion of Physical Culture<br>(Towarzystwo Krzewienia Kultury Fizycznej)	Association promoting physical culture established on May 3, 1957 in Warsaw. It has been operating on regional and local levels mainly although this is a national association. It's aim is to promote and set up initiatives enhancing active tourism and physical culture.<br>Web page: https://pl.wikipedia.org/wiki/Towarzystwo_Krzewienia_Kultury_Fizycznej
Polish Society of Sports Medicine<br>(Polskie Towarzystwo Medycyny Sportowej)	PTMS is a scientific and educational type of organization. The goals of the Society include mainly: providing inspiration for scientific research, promoting knowledge of sports medicine, cooperation in increasing occupational skills and offering assistance to society members in their scientific and occupational activities.<br>Polish Society of Sports Medicine has developed e.g. specific recommendations on physical activity for adults and older adults, based on recommendations of the European Society of Cardiology, the American College of Sports Medicine and the American Heart Association.<br>Web page: http://en.ptms.org.pl/
Association of Healthy Polish Cities<br>(Stowarzyszenie Zdrowych Miast Polskich)	The aim of the Association is to implement health policy principles and strategies in the country, regions and municipalities, As part of the tasks, the Association inspires and supports local programs of member cities in the field of health protection, develops cooperation between cities and municipalities located in regions with high environmental pollution, and cooperates with institutions and organizations dealing with health and environmental issues.
Association of Polish Poviats<br>(Związek Powiatów Polskich)	The aim of the association is initiating and giving opinions on draft legal acts concerning local self-governments, promoting the exchange of experience in the scope of own tasks and tasks in the field of government administration, inspiring and undertaking joint initiatives affecting the development of poviats. The association is very active within the area of sports and physical activity.
Polish cardiac society<br>(Polskie Towarzystwo Kardiologiczne)	The aim of the society is prevention and control of heart and vessel diseases as well as promotion and protection health in the field of cardiology and medicine related to it among society.
Polish Prevention Forum<br>(Polskie Forum Profilaktyki)	The PFP's objectivies are:<br>- exchange of information and harmonisation of guidelines<br>prophylaxis in Poland,<br>- adapting the principles of European prevention to the conditions and specificity of the country,<br>- developing a common promotion and information system on epidemiology and disease prevention approaches<br>of the cardiovascular system,<br>- coordination of activities in the field of prevention and education at national level,<br>- preparation and implementation of a curriculum on epidemiology and prevention of cardiovascular diseases in primary schools, junior high schools, high schools and other secondary schools.
Polish Federation of Food Industry<br>*(Polska Federacja Żywności)*	The main objective of the functioning of the Scientific Council of PFPZ is a exchanging information between the science world and wthe food industry on the latest trend, solutions, technologies and researchers. The Employers' Assocations of the PFPZ is working on the development of food industry and strengthening its representation in Poland and in EU, by conducting and supporting initiatives in the area of corporate social responsibility, with particular emphasis on consumer education in the field of food, nutrition and labelling of food products.PFPZ is also actively involved in the „Keep fit programme".<br>Web page: https://www.pfpz.pl/index/?lang_id=2
The University Sports Association	The University Sports Association's objectivies include:<br>- promotion of sport, physical culture and tourism, improvement of physical fitness and health of the academic community,

(Akademicki Związek Sportowy)	- development of competitive sport in the academic environment,<br>- promotion of physical culture and sport and rehabilitation of people with disabilities,<br>-promotion and dissemination of the principles of Olympism, ideas and Olympic education, fair play sports attitudes,<br>-action on health promotion and health education adapted to the needs of different social groups.<br>Web page: http://azs.pl/english/azs-what-is-it
Polish National Food and Nutriition Institute (Insytut Żywności i Żywienia)	The main objective of the functioning of the Institute is improvement of the public the awareness and knowledge of food, physical activity and their impact on health.<br>Web page: http://www.izz.waw.pl

SECTION 2
Leadership and partnerships

Questions 2 and 3 are about the situation relating to **leadership** and **coordination** of efforts to promote physical activity. In this context, the terms are used as follows.

- Leadership refers to the provision of overall direction for HEPA; e.g. responsibility for defining, supervising and managing the national physical activity agenda.
- Coordination means communication on, and alignment of actions and developments relating to HEPA, and could include facilitation of regular exchange between relevant stakeholders.

Leadership and coordination can be provided by one or more agencies within or outside of government. The same or different agencies may be involved in both activities, and different agencies might be involved at national and subnational levels. Mechanisms for leadership and coordination can take the form of a multisectoral committee, working group, alliance or task force, or might be led by a government agency or NGO

2. Please state any agency(ies) providing **leadership for HEPA promotion** at the **national level** in your country.
Ministry of Sport and Tourism – HEPA focal point

3. Please state any agency(ies) providing **leadership for HEPA promotion at the subnational level** (e.g. at regional/ provincial/cantonal/municipal level) in your country.
There is no one (although there is a recommendation to create it in Poland).

4. Are any mechanisms or agencies in place in your country to ensure **cross-sectoral collaboration** on the delivery of HEPA policy, **at the national level**?<br>**If yes,** briefly describe. Please provide information on who is involved, who is leading these efforts, and how these collaborations function in practice. Please also mention (to the extent possible) any positive or more difficult experiences. This may also include examples of collaboration with the private and voluntary sectors.
No, there are neither mechanisms nor agencies to ensure the collaboration. The only bodies that could take the responsibility could be the Public Health Council (Rada ds. Zdrowia Publicznego) and Steering Committee of National Health Program for 2016-2020(NPZ 2016-2020). In reality these bodies are not active.

5. Are any mechanisms or bodies in place in your country to ensure **cross-sectoral collaboration** on the delivery of HEPA policy **at the subnational level**?<br>**If yes,** briefly describe. Please provide information on who is involved, who is leading these efforts, how these collaborations function in practice. Please also mention (to the extent possible) any positive or more difficult experiences. This may also include examples of collaboration with the private and voluntary sectors.
No, although there are cases in single towns at the local level (Mrągowo, Lidzbark Warmiński) where this kind of bodies were created (e.g. to examine faulty postures among children)

SECTION 3
Policy documents

Question 6 is about **any relevant past policy documents or past events** that were influential in shaping the HEPA agenda in your country. This will provide background context about HEPA in your country. **Current policy documents** are described in Question 7.

6. Please describe any **key past policy documents** and **past events** that have led to the current context of HEPA promotion in your country. This might include legislation or recent policy documents that are now technically out of date (e.g. a previous national HEPA policy that may or may not have been extended), previous landmark legislation, or other documents such as scientific reports. Key events might include political changes, position statements or scientific events that have shaped the HEPA agenda.<br>Please list the documents/events, provide a web link (where available), and indicate if an English version or summary is available in each case.<br>Please add/remove rows as needed.
**Key past documents** (please repeat as needed)<br>**Policy title:** *The National Health Program 2007-2015*<br>Publication date: 2007<br>Time frame covered (if specified): 2007-2015<br>Issuing body: Ministry of Health<br>Web link (English version not available):<br>http://www2.mz.gov.pl/wwwfiles/ma_struktura/docs/zal_urm_npz_90_15052007p.pdf<br>Description: The National Health Program 2007-2015 is a Polish public health policy aiming to improve health and related quality of life of the population and reducing inequities in health achieved by: shaping a pro-health lifestyle of the society as well as creating a living environment, work and health-promoting learning, activating local government units and organizations non-governmental organizations for health activities.<br>It consists out of 8 strategic and 15 operational goals. Four of the operational goals refer to PA:<br>Operational goal 4: Increasing physical activity of the population<br>Operational goal 8: Supporting physical and psychosocial development and health as well as prevention the most common health and social problems of children and adolescents<br>Operational goal 9: Creating conditions for a healthy and active life of older people<br>Operational goal 10: Creating conditions for the active life of people with disabilities.
**Policy title:** *National neoplasmatic (cancer) disease program for 2006-2015*<br>Publication date: December 2015<br>Time frame covered (if specified): 2006-2015<br>Issuing body: Ministry of Health<br>Web link (English version not available):

http://www.archiwum.mz.gov.pl/zdrowie-i-profilaktyka/narodowy-program-zwalczania-chorob-nowotworowych/narodowy-program-zwalczania-chorob-nowotworowych-na-lata-2006-2015/<br>Description: The program is primarily aimed at preventing cancer disease. The program consists out of 5 priorities. The first one is "Health promotion and cancer prevention". Information and educational activities constitute a priority in the first task. Increasing awareness and improving the quality of life of children and youth through creating the conditions and motivating, knowledge and skills for influence healthy lifestyles for cancer prevention; attitude building physical activity and healthy lifestyle;
**Policy title:** *Sport development strategy until 2015*<br>Publication date: January 2007<br>Time frame covered (if specified): 2007-2015<br>Issuing body: Ministry of National Education and Sports<br>Web link (English version not available):<br>http://www.delf.home.pl/regulaminy/strategia2015.pdf<br>Description: The main objective of the programme is an active and efficient society. The strategy consists of three priorities. The first priority concerns the promotion of sport for all. The objective of the first priority is to achieve an optimal level of behavioural consolidation pro-health society in different age groups, environmental groups. The first priority focuses of two areas:<br>- physical fitness of children and young people,<br>- physical activity of the society.
**Policy title:** *Directions of tourism development until 2015*<br>Publication date: September 2008<br>Time frame covered (if specified): no<br>Issuing body: Ministry of Sport and Tourism<br>Web link (English version not available): https://bip.kprm.gov.pl/kpr/wykaz/r1332,Program-Rozwoju-Turystyki-do-2020-roku.html<br>Description:<br>The main objective of the Program is strengthening the development of competitive and innovative tourism by supporting enterprises, organizations and institutions and tourism sector initiatives. This goal will be implemented through four operational goals:<br>1. Development of innovativeness, attractiveness, quality of services and tourist products as a competitive factor of the economy.<br>2. Strengthening social activity and entrepreneurship in the tourism economy sector and increasing the competences of human resources in managing tourism entrepreneurship.<br>3. Promotion of branded tourist products of the country and regions as well as economic specializations based on tourism.<br>4. Development and modernization of space for the development of tourism and tourism development infrastructure while maintaining environmental protection and applying eco-innovative technologies.<br>Objective number 4 directly refers to the support of the physical activity development.
**Key past events**
7. Please provide details (title, timeframe, issuing body) of the **current key policy documents, legislation, strategies or action plans** in your country, which outline government (and, where applicable, NGO) intention to increase national levels of physical activity (see Glossary for definitions of these terms).<br>Please list the documents according to sector and, where available, provide a web link, indicating whether an English version or summary is available. Please provide a brief description of the general content of each policy (about 100–250 words).<br>Please mark in the right-hand column **which are the most important documents** for the HEPA agenda in your country and briefly explain why these documents are deemed important.<br>Please add/remove rows as needed.

Sector	Policy	Indicate (X) most important documents
Example: Health	**Policy title: Resolution on the development of health enhancing physical activity and diet** (Valtioneuvoston periaatepäätös terveyttä edistävän liikunnan ja ravinnon kehittämislinjoista)<br>Publication date: 2008<br>Time frame covered (if specified): 2008–2011<br>Issuing body: Ministry of Health and Social Affairs<br>Web link: English version available at:<br>https://julkaisut.valtioneuvosto.fi/<br>Description: The statement is the main current political document on HEPA, providing the political and government strategy for physical activity in Finland. This resolution is multisectoral in nature, and was developed mainly by the Finnish Ministry of Health and Social Affairs in collaboration with the Ministry Education and Culture. It contains specific population targets and proposes principal methods for enhancing HEPA and healthy diet:<br>- within different populations (children, young people, families, students, workers, older people);<br>- through the local environment (including equal access)<br>- through political decisions at the local level<br>- through education (doctors, nurses, teachers, etc.)<br>- through national surveys and follow-up.<br>The last chapter emphasizes how the resolution is translated into action, and how it will be followed up.	*X*
Health	**Policy title:** *The National Health Program 2016-2020*<br>Publication date: August 2016<br>Time frame covered (if specified): 2016-2020<br>Issuing body: Ministry of Health<br>Web link (English version not available):<br>https://www.gov.pl/web/zdrowie/npz-2016-2020<br>Description: The National Health Program is a basic document of Polish public health policy that determines strategic objectives, operational goals and the most important tasks to be carried out in order to improve health and the associated quality of life in society. It consists of 6 operational goals. Two of them directly refer to PA:<br>Operational goal 1: Improving the diet, nutritional status and physical activity of the society<br>Operational goal 5: Promotion of healthy and active aging.	
	**Policy title:** *National neoplasmatic (cancer) disease program*<br>Publication date: December 2015<br>Time frame covered (if specified): 2016-2024<br>Issuing body: Ministry of Health<br>Web link (English version not available):<br>http://www.archiwum.mz.gov.pl/wp-content/uploads/2015/12/Uchwala-Nr-208-NPZCHN-2016-2024.pdf<br>Description: The program is primarily aimed at preventing cancer disease. Activities focus on increasing health awareness and healthy lifestyle through promotion and health education. In connection with demographic challenges in the next decade, the emphasis will be on those segments of health protection that correspond to the epidemiological challenges of an aging population. The program consists out of 5 priorities. The first one is “Health promotion and cancer prevention” and it aims at increasing the awareness of society and its education and will be implemented both at the central and local level consisting mainly of information, promotional and educational activities activities promoting active lifestyle.	

Sector	Policy	Indicate (X) most important documents
Health	**Policy title:** *Program for the prevention and treatment of cardiovascular disease POLKARD 2017-2020*<br>Publication date: July 2017<br>Time frame covered (if specified): 2017-2020<br>Issuing body: Ministry of Health<br>Web link (English version not available):<br>http://www.archiwum.mz.gov.pl/wp-content/uploads/2013/12/polkard-2017-2020-przyjety-do-realizacji-06072017.pdf<br>Description: The main goal of the program is to reduce mortality due to heart and vascular<br>diseases in Poland. The program has six specific objectives. The objective number 2 aims at conducting nationwide primary prevention of diseases of the cardiovascular system and refers directly to physical activity. As part of the task, it is planned to implement following subtasks:<br>- health education of the society through an informational and educational campaign in the field of prevention and prevention of cardiovascular diseases (one of the proposed intervention is popularization of physical activity and promotion of healthy and safe physical training and effort adapted for various age groups as well as patients with heart and vessel diseases<br>- educational and intervention program for cardiovascular risk reduction for school children and adolescents<br>- development and implementation of an effective method in the field of high-risk strategies including primary prevention among adults.	
Sport and recreation	**Policy title:** *Sport development programme 2020*<br>Publication date: April, 2015<br>Time frame covered (if specified): 2015-2020<br>Issuing body: Ministry of Sport and Tourism<br>Web link (English version):<br>https://www.msit.gov.pl/download/3/12543/prssummaryang74e4.pdf<br>Description: The main objective provided for in the document is to create conditions that will enable progress towards the presented vision i.e. towards the ideal situation of society being active and healthy. The main objective involves two key components: ensuring the conditions for the development of physical activity (sports infrastructure and offers, as well as appropriate organizational structures), and promoting a healthy and active lifestyle.<br>The main objective will be achieved through specific objectives related to:<br>- ensuring the appropriate conditions and offers for practicing physical activity at each stage of life,<br>- using the potential of sport to build social capital,<br>- improving the organizational and legislative conditions for the development of sport, and increasing the availability of qualified human resources,<br>using the potential of competitive sport for popularizing physical activity and for promoting Poland internationally.	
	**Policy title:** *Tourism Development Programme until 2020*<br>Publication date: August 2015<br>Time frame covered: 2015-2020<br>Issuing body: Ministry of Sport and Tourism<br>Web link (English version available):<br>https://www.msit.gov.pl/download/3/12550/TourismDevelopmentProgrammeuntil20201f3c.pdf	

Sector	Policy	Indicate (X) most important documents
	Description: This program is a continuation of the former program: *Directions of tourism development until 2015* with the same aims and scope of activities proposed. The main objective is strengthening the development of competitive and innovative tourism by supporting enterprises, organizations and institutions and tourism sector initiatives (see Quest. No. 6)	
Education	**Policy titile:** *Journal of Law 2019, item 639, Regulation of the Minister of National Education of 3 April 2012 on framework teaching plans for public schools*<br>Publication date: April 2012<br>Time frame covered (if specified): no timeframe<br>Issuing body: Ministry od National Education<br>Web link (English version not available): http://prawo.sejm.gov.pl/isap.nsf/download.xsp/WDU20190000639/O/D20190639.pdf<br>Description: The Regulation is a basic document of Polish education policy. It describes a framework teaching plans for public schools for example a weekly hours for physical education. According to the Law physical education is mandatory up to have:<br>- 4 hours per week in primary school<br>- 3 hours per week in secondary school<br>- 3 hours per week in special needs schools.	
	**Policy title:** *Journal of Law 2018, item 1668, Act of 20 July 2018, The Law on Higher Education and Science*<br>Publication date: July 2018<br>Time frame covered (if specified): no timeframe<br>Issuing body: Parliament<br>Web link (English version not available): http://prawo.sejm.gov.pl/isap.nsf/download.xsp/WDU20180001668/T/D20181668L.pdf<br>Description: The Act is a basic document for the functioning of the higher education and science in Poland. The section IX of the Act presents the way of promotion sporting achievements (a rector's scholarship and the ministers scholarship). The rector's scholarship for an outstanding student may be awarded student who has been highly placed in international or national sport contests. The minister's scholarship for academic achievement of outstanding sporting achievements.	
Transport	**Policy title:** *Transport Development Strategy until 2020*<br>Publication date: January 2013<br>Time frame covered: 2013-2020 (with perpective of 2030)<br>Issuing body: Ministry of Infrastructure (former Ministry of Transport, Construction and Maritime Economy)<br>Web link (English version not available): https://www.gov.pl/documents/905843/1047987/Strategia_Rozwoju_Transportu_do_2020_roku.pdf/ead3114a-aac7-3cdd-c71d-7f88267ce596<br>Description: The main purpose of national transport policy is to increase territorial accessibility and improving the safety of road users and the efficiency of the transport sector through creation of a coherent, sustainable and user-friendly transport system in national (local), European and global dimension. The issues of physical activity can be found:<br>- under objective 4: Integrated transport system in Poland,<br>- detailed objective 4.5: urban transport as an element of the integrated transport system,	

Sector	Policy	Indicate (X) most important documents
	and actions: to ensure a balance between transport capacity to serve economic development, respect for the natural environment and maintaining the quality of life in the future, as well as: promoting walking and cycling.	
	**Policy title:** *Transport Development Strategy until 2030*<br>Publication date: Septemnber 2019<br>Time frame covered: 2020-2030<br>Issuing body: Ministry of Infrastructure (former Ministry of Transport, Construction and Maritime Economy)<br>Web link (English version not available):<br>https://www.gov.pl/web/infrastruktura/projekt-strategii-zrownowazonego-rozwoju-transportu-do-2030-roku2<br>Description: The main objective of the national transport policy presented in the strategy is to increase the country's transport accessibility and improve the safety of traffic participants and the efficiency of the transport sector by creating a coherent, sustainable, innovative and user-friendly transport system at the national, European and global levels. The priority to improve urban transport management is to reduce congestion in cities. and their functional areas, inter alia by increasing the share of public transport in passenger transport, promotion of eco-mobility chains - cycling and walking.	
	**Policy title:** *National Strategy for Regional Development 2030 Draft.*<br>Publication date: September 2019<br>Time frame covered: 2020-2030<br>Issuing body: Ministry of Investment and Development<br>Web link (English version not available):<br>https://www.gov.pl/attachment/38c54257-5b35-4b2d-b379-c897a31c85e7<br>Description: The main area of impact of regional policy in the field of transport is support for transport infrastructure actions aimed at improving the accessibility of the Polish space. In the following areas rural areas, measures will be implemented within the scope of construction and odernisation of basic transport infrastructure (local roads, associated infrastructure, cycle paths) and the development of services and means of transport.	
Environ-ment	**Policy title:** *National Strategy for Regional Development 2030*<br>Publication date: September 2019<br>Time frame covered: 2020-2030<br>Issuing body: Ministry of Investment and Development<br>Web link (English version not available):<br>https://www.gov.pl/web/inwestycje-rozwoj/krajowa-strategia-rozwoju-regionalnego<br>Description: The document defines the regional policy of the state in the perspective to 2030. In order to ensure the highest possible efficiency and effectiveness of the activities carried out in the National Strategy, it is necessary to be guided by specific regional policy principles. Principle 6 concerns sustainable investment. An example of practical application of the principle is the cooperation of municipalities in the field of joint spatial planning, e.g. organisation of cycling and walking opportunities in city centres is a part of this policy.	
	**Policy title:** *Strategy for sustainable development of rural areas, agriculture and fisheries until 2030*<br>Publication date: 2019<br>Time frame covered: 2019-2030<br>Issuing body: The Ministry of Agriculture and Rural Development	

Sector	Policy	Indicate (X) most important documents
	Web link (English version not available): http://www.kowr.gov.pl/biuro-prasowe/aktualnosci/strategia-zrownowazonego-rozwoju-wsi-rolnictwa-i-rybactwa-2030<br>Description: The long-term objective of the strategy is development of rural areas, agriculture and fisheries by improving the quality of life in rural areas and effective use of their resources and potentials, including agriculture and fisheries, for sustainable development of the country. The pursuit of the main objective referring to HEPA will be realized through actions assigned to following objectives:<br>Objective 2. Improvement of living conditions, infrastructure and evironment;<br>Objective 3. Development of entrepreneurship, non-agricultural jobs and active society.<br><br>Strategy activities meet new civilization challenges, including such as: aging populations, climate change, generational change, information technology development, occupational and territorial mobility, and the impact of the demographic situation in the world on food security.	
Urban design and planning	**Policy title:** *National Urban Policy 2023*<br>Publication date: October 2015<br>Time frame covered: 2015-2023<br>Issuing body: Ministry of Infrastructure and Development<br>Web link (English version not available):<br>http://prawo.sejm.gov.pl/isap.nsf/download.xsp/WMP20150001235/O/M20151235.pdf<br>Description: Priority of pedestrian and bicycle transport (and public transport), in particular in city centers, is one of the directions set out in the National Urban Policy. There is a need to create an appropriate infrastructure and a friendly urban space that would encourage city residents to go hiking or cycling. The National Urban Policy recommends supporting activities leading to the perception of a bicycle as an important means of transport which is to be implemented by creating a comprehensive bicycle infrastructure in the form of e.g. separate bicycle routes, places and devices for parking bicycles using a comprehensive approach including the elimination of even small aspects that may discourage the use of bicycles. Also pedestrian traffic must be treated as an equal way of moving. In spatial and communication solutions, it is necessary to take into account the needs of pedestrians - both in downtown areas and outside them - by shortening the access roads to a minimum, installing amenities and eliminating barriers.	
Other sector (Social policy)	**Policy title:** *Long-Term Senior Policy in Poland for years 2014-2020* (transformed into *Multi-Annual Programme Senior + for 2015–2020)*<br>Publication date: December 2013<br>Time frame covered: 2014-2020<br>Issuing body: Ministry of Family, Labour and Social Policy<br>Web link (English version not available):<br>https://das.mpips.gov.pl/source/Long-term%20Senior%20Policy.pdf and http://prawo.sejm.gov.pl/isap.nsf/download.xsp/WMP20160001254/O/M20161254.pdf<br>Description: In order to take the issue of ageing into account in all policy areas to adjust societies and economies to demographic change and to create a society open to all age groups in Poland, public policy framework with regard to older people have been adopted. The aim of the senior policy in Poland is support and provide opportunities for active ageing in	

Sector	Policy	Indicate (X) the most important documents
	health and opportunities for continued self-reliant, independent and fulfilling life, even with some functional limitations.<br>Within the area of physical activity the program has got two objectives:<br>Objective 1: Promoting appropriate lifestyle<br>Objective 2: : Development and promotion of physical activity<br>The recommended interventions includes: to increase the availability of existing sports facilities for the elderly, the development of an environment favorable to seniors physical activity, such as place of residence, in the workplace, in public places, promotion of the initiatives concerning the physical activity amongst elderly people, development and support a system of organized physical activity classes for adults and older.<br>Contact: Department of Senior Policy, Ministry of Family, Labour and Social Policy	

8. During the development of the most important policies/action plans listed in Question 7, was a **consultative process** used, involving relevant stakeholders?<br>**If yes**, please briefly outline the steps of this consultation processes and which organizations were involved. Please also mention any challenges in recent years in engaging government ministries or other agencies through such processes.
Every public document goes through the process of social consultations. Besides, strategic documents e.g. all draft resolutions of the Council of Ministers must undergo inter-ministerial and social consultations. Conferences and workshops are organised to consult drafts of public documents. Additional opinions are collected in paper and/or electronic form. In consultation procesess are involved regional or local authorities, scientific and academic communities etc.

9. In your appraisal of the policy documents listed in Question 7, is there evidence of **cross-referencing and alignment within and between policies, with genuine connections between different policy areas**, or do the policies present separate, sector-specific strategies without evidence of links and consistency across sectors and documents with relevant policy?<br>For example: in the health sector, does a national obesity prevention strategy refer to an existing physical activity promotion plan, thus demonstrating an integrated overarching national approach to addressing physical activity? Does a transport policy recognize links with other policies that promote walking and cycling in the health sector (or sport field)? Does a sport promotion policy cross-reference HEPA promotion activities contained in a health promotion policy?<br>**If yes**, please briefly explain and give examples of such cross-referencing. Please state which of the policy documents presented in Question 7 you are referring to.
No, there is no very evident reference between Polish policies. The main connections can be seen in reference to European policies. E.g. within the sport area. *Sport development programme 2020 refers treats* the European Commission's White Paper on Sport as the basic document that sets out strategic guidelines for the role of sport in Europe and Poland as well.

10. In your country, are any mechanisms in place to ensure that the key policy documents listed in Question 7 are **based on the best-available scientific evidence on HEPA**?<br>For example, are specific mechanisms or agencies dedicated to reviewing evidence and ensuring that the latest evidence is used to inform national policy development? Do any formal committees or institutions exist that are responsible for reviewing evidence and providing guidance to national policy-making bodies, or any formal links between government and academic institutions for this purpose?<br>**If yes**, please briefly describe these.

No formal links but both Ministry of Health and Ministry of Sport and Tourism has establishes unformal contacts with institutes and research centres who provide them with facts, figures and knowledge regarding sport and health monitoring and supporting initiatives. If it is necessary to order a specific study or expertise, the ministries use the formal competition procedure - public procurement (offer inquiry).<br>The institutes who cooperate with the ministries are e.g.: Sports Institute National Research Institute, University of Phisical Education in Warsaw, National Institute of Public Health – National Institute of Hygiene, University of Warsaw.

11. Please indicate **how useful the following international documents have been** in the development of physical activity- related policy in your country, e.g. by serving as a basis, input or inspiration (whether having been specifically quoted or not in a policy document). Please rate the documents below on the scale from 1 (= "not at all useful") to 5 (= "very useful"). Please add any other international documents which have been important in the development of physical activity-related policy in your country, as necessary.

	Not at all useful				Very useful	
	1	2	3	4	5	Don't know
Global strategy on diet, physical activity and health (2)					X	
Global recommendations on physical activity for health (3)					X	
2008–2013 action plan for the global strategy for the prevention and control of noncommunicable diseases (4)					X	
Global status report on noncommunicable diseases 2010 (5)					X	
Global action plan for the prevention and control of noncommunicable diseases 2013–2020 (6)					X	
Steps to health. A European framework to promote physical activity for health (7)					X	
Action plan for implementation of the European strategy for the prevention and control of noncommunicable diseases 2012−2016 (8)					X	
The Toronto Charter for physical activity: a global call for action (9)		X				
Noncommunicable disease prevention: investments that work for physical activity (10)		X				
Lancet series on Physical Activity (11)		X				
Other document (please specify): WHITE PAPER ON SPORT https://eur-lex.europa.eu/legal-content/EN/TXT/PDF/?uri=CELEX:52007DC0391&from=PL					X	
Other document (please specify): EU Physical Activity Guidelines https://eacea.ec.europa.eu/sites/eacea-site/files/eu-physical-activity-guidelines-2008.pdf					X	
Other document (please specify): *Communication: Developing the European Dimension in Sport* https://eurlex.europa.eu/LexUriServ/LexUriServ.do?uri=COM:2011:0012:FIN:EN:PDF					X	

12. Do any **national documents or guidelines** exist that **support implementation of HEPA activities at the subnational level**? For example, does national policy determine what is delivered at the subnational level and, if so, is this national guidance strongly adhered to? Such guidance could include programmes, structures or funding. Or is subnational policy and activity developed and implemented largely independently from the national government?<br>**Please note:** please be brief here (about 300–500 words) and include cross-references to other questions (e.g. Question 7) where relevant, to avoid repetition.
Sport Development Programme 2020 supports implementation of HEPA activities at the subnational level by realization and financing of two programs: *Sport for All Program* and *Sport for All Children*.<br>https://bip.msit.gov.pl/download/2/5611/ProgramRozwojuSportudoroku2020-wersjazdnia6lipca2015.pdf<br>The "Sport for All" program is implemented as part of promoting sport in society by supporting projects in the field of promoting sport in various social and environmental groups. Every year, the Minister of Sport and Tourism announces an open competition to co-finance (from the state budget) tasks related to sport from various social and environmental groups. Non-governmental organizations established to carry out tasks related to sport and physical culture may apply for funding as part of the competition. In particular, these are nationwide entities with appropriate (supra-regional and voivodship) organizational structures, i.e. associations, foundations. The amount of PLN 20 260 000 was allocated to the implementation of in 2016. The amount of PLN 22 562 000 was allocated in 2017. In 2018, PLN 24,000,000 was allocated for the implementation of the programme. In 2019 321 applications were approved and the financial means in the form of grants for the total amount of PLN 20,934,500 were approved.<br>“Sport for All Children” program is implemented in a similar way. Every year, the Minister of Sport and Tourism announces an open competition to co-finance (from the state budget) tasks related to developing sport by supporting children's and youth sport and physical activity. In 2019 the amount of financial support was PLN 54,500,000.

SECTION 4
Policy scope, content and implementation

13. Considering all the key physical activity policy documents listed in Question 7, please indicate which **settings are included for the delivery of specific HEPA actions**.<br>Please only tick those settings in which dedicated programmes or interventions are foreseen or already under way.			
Preschools/kindergarten		Sport and recreation	
Primary schools		Transport	
Secondary/high schools		Tourism	
Colleges/universities		Environment	
Primary health care		Urban design and planning	
Clinical health care (e.g. hospitals)		Community	
Workplace		Other (please specify):	
Older adult/senior services			

14. Considering all the key physical activity policy documents listed in Question 7, please indicate which **population groups are targeted by specific HEPA actions**.<br>Please only tick those groups for which dedicated programmes or interventions are foreseen or already under way.			
Early years	X	Sedentary/the least active	X

Children/young people	X	People from low socio-economic status	X
Older adults	X	Families	X
Workforce/employees		Indigenous people	
Women	X	Migrant populations	X
People with disabilities	X	General population	
Clinical populations/chronic disease patients	X	Other (please specify):	

15. Does your country have a current **national communication strategy (using mass media)** aimed at raising awareness and promoting physical activity?<br>**If yes**, please provide details of the communication activities (e.g. posters, website, television or radio advertising, etc.) and whether these activities have a common branding or slogan (e.g. "Agita Sao Paulo" or "Find 30").<br>**If no**, has your country conducted any national communication activities in the past?
No, but they finance some other initiatives, e.g. social media canals (600 events per year on Tweeter and Facebook)

16. To illustrate the types of policy actions in your country, please provide one or two **examples** (if available) of large-scale (preferably national) programmes or interventions in each of the settings listed.<br>Please provide a brief description of each programme or intervention (about 100 words, including, for example: name, lead organization, approach, participants, results.) and a source where further information can be obtained.<br>Suggestion: You could also consider developing these examples into more detailed case studies to complement your national PAT assessment.	
Example: sport/recreation	Youth and Sports is the Swiss national sports promotion programme for people aged 5–20 years. It offers courses in more than 70 disciplines and reaches more than half a million children and adolescents every year. More recently, it also offers sports promotion activities for children aged 5–10 years. Youth and Sports is based on the Federal Law on the Promotion of Gymnastics and Sport (described above).<br>More information: Federal Office of Sport website (German, French, Italian) (http://www.jugendundsport.ch/) (12) and Kelly P, Cavill N, Foster C. An analysis of national approaches to promoting physical activity and sports in children and adolescents. Full report. Oxford: University of Oxford British Heart Foundation Health Promotion Research Group: 2009 (www.euro.who.int/__data/assets/pdf_file/0009/119295/HEPA_children_analysis_report.pdf).
Health	*"Keep Balance - Prevention of overweight, obesity as well as chronic diseases by education on nutrition and physical activity of the society project"*<br><br>The main objective of the project is to promote healthy lifestyles, including the prevention of overweight and obesity, and other chronic non-communicable diseases through education in nutrition and physical activity.<br><br>Web page: http://www.zachowajrownowage.pl
Sport/recreation	*The National Talent Base*<br><br>The National Talent Base is a simple pubic tool for monitoring and testing the level of physical fitness of young people. On the basis of measurements and results of physical fitness tests it is possible to compare the efficiency of participants in relation to the population and to search for people with above-average results.<br><br>Web page: https://www.narodowabazatalentow.pl

	Local Sports Animator<br><br>The Local Sports Animator is a systemic project which main objective is to promote physical activity and sport among children and youth by enabling them to participate in extracurricular and out-of-school sports activities, co-financed from the Fund for Physical Development, which is administered by the Ministry of Sport and Tourism.<br><br>Web page: http://www.orlysportu.pl
	All Children's Sport<br><br>Sport All Children supports financially projects in the field of promotion of sport for children and youth. In three tasks:<br>- The "Local Sports Animator" project (recruitment for the national operator)<br>- Supporting the organisation of sporting events for children and young people<br>- Supporting projects promoting the sport of children and youth implemented by Polish sports associations<br><br>Web page: https://bip.msit.gov.pl/bip/finansowanie-zadan/sport-powszechny/2019-rok/2553,Program-Sport-dla-Wszystkich-w-ramach-upowszechniania-sportu-w-spoleczenstwie-po.html
	Sports for All<br><br>The objectives of the programme are:<br>- to increase physical activity of the society in various social and environmental groups;<br>- creating conditions for active leisure, shaping a healthy lifestyle;<br>- supporting initiatives and activities for the development of sport's activities and dissemination of good practices throughout the country;<br>- promoting healthy, social, educational and educational values of sport;<br>- counteracting civilization diseases (including overweight and obesity);<br><br>Web page: https://bip.msit.gov.pl/bip/finansowanie-zadan/sport-powszechny/2019-rok/2553,Program-Sport-dla-Wszystkich-w-ramach-upowszechniania-sportu-w-spoleczenstwie-po.html
Education	*Stop abstention from PE classes*<br><br>This scheme was established in 2013 by the Ministry of Sport and Tourism. The main goal of the campaign is to promote physical activity among schoolchildren and adolescents and to encourage them to attend physical education (PE) classes at school. The target groups include children and adolescents themselves, along with parents, PE teachers, schools, local governments and doctors<br>Web page: https://www.msit.gov.pl/pl/aktualnosci/6889,STOP-ZWOLNIENIOM-Z-WF-u.html
	School Sports Club Programme<br>*(Szkoly Klub Sportowy)*<br><br>The School Sports Club Programme is a systemic activity addressed to primary and secondary school students. The aim of the SKS are :<br>- stimulating and shaping the habit of physical activity among children and young people,<br>- improve physical fitness, health and quality of life of children and young people, including the prevention of overweight and obesity,<br>- promotion of a healthy and active lifestyle among children and young people,

	- increasing the offer of generally accessible, free sport and recreation activities for children and youth,<br>- motivation to undertake physical activity among less active and physically fit children and adolescents, with particular emphasis on girls,<br>- promotion of various sports among children and youth (etc.).<br>Web page: https://www.szkolnyklubsportowy.pl/
	Major National: "Keep fit!" Educational Programme<br><br>The objective of the "Keep Fit!" Programme was to educate about permanent forging of healthy habits of the school adolescents (secondary schools) through the promotion of the principles of active lifestyle and balanced diet, based on individual responsibility and free selection of individual<br><br>The main objective will be achieved through specific objectives related to:<br>- forging of healthy habits concerned with lifestyle and nutritional pattern,<br>- encouragement of physical activity (both during school classes and recreational activities,<br>- supply of up-to-date knowledge about nutritional patterns with special focus of diet diversification and balancing both in energy and nutritional terms.<br>Web page: http://www.trzymajforme.pl/
Transport	*Cycling May*<br><br>The aim of Cycling May is to promote a healthy lifestyle and sustainable mobility among preschool children, primary school students and teachers. Cycling May, through fun combined with elements of competition, promotes cycling as a means of transport to school, teaches good and healthy habits, which are maintained even after the end of the campaign. Cycling May effectively changes the surroundings of schools and kindergartens to more safe and cyclist-friendly by reducing the number of cars carrying children and motivating local governments to make pro-cycling investments.<br>Web page: https://rowerowymaj.eu/
Environment	*Spin Kilometres*<br><br>Campaign promote bicycles as the most eco-friendly means of transport, develop the bicycle infrastructure, encourage to lead a healthy lifestyle, and stimulate discussion. Spin Kilometres is a competition between cities for "the most bicycle friendly city" title. During the six years of the campaign (2012-2018) the participants travelled a total of 190 687 167 km, which has reduced 49 612 tone of CO_2<br><br>Web page: https://raportcsr.allegro.pl/en/social/fundation and https://kreckilometry.pl/o-kampanii

SECTION 5 Recommendations, goals and targets

This section contains questions referring to national recommendations on physical activity (Question 17a) and sedentary behaviour (Question 17b).

17a. Does your country have any **national recommendations on physical activity and health**? National recommendations refer to a consensus statement on how much activity is required for health benefits. **If recommendations exist for any of the target groups listed,** please provide details for the population subgroups (where applicable), including issuing body, year of publication, title of the document, and provide a web link if available (please also specify whether the document is available in English). **If no recommendations exist, please mark the "no" column for the respective target group.** If your country has officially adopted or endorsed international recommendations (e.g. of WHO or the United States Department of Health), this should be mentioned as part of the description of the respective recommendations.		
		**No**
Early years (pre-school age)	Document/policy title and date:<br>National recommendations for healthy physical activity, August 2018<br>Issuing body: Ministry of Sport and Tourism<br>Web link (English version not available):<br>https://www.awf.katowice.pl/sites/default/files/uploads/Newsy/2018/raport_str_tyt.png<br>Briefly state the recommendations and specify age range:<br>0-12 months - allow the child freedom of movement and stimulus<br>1-3 year - create the opportunity for children to meet their individual needs and encourage them to run, jump, and throw<br>4-6/7 years – min. 180 min.of PA per day, opt. more than 180 min.	
Children and young people (school-age)	Document/policy title and date: National recommendations for healthy activity, August 2018<br>Issuing body: Ministry of Sport and Tourism<br>Web link (English version not available):<br>https://www.awf.katowice.pl/sites/default/files/uploads/Newsy/2018/raport_str_tyt.png<br>Briefly state the recommendations and specify age range:<br>6/7-11 years – min. 60 min.of PA per day, opti. more than 60 min.<br>11/12-17 years – min. 60 min.of PA per day, opti. more than 60 min.	
Adults	Document/policy title and date: National recommendations for healthy activity, August 2018<br>Issuing body: Ministry of Sport and Tourism<br>Web link (English version not available):<br>https://www.awf.katowice.pl/sites/default/files/uploads/Newsy/2018/raport_str_tyt.png<br>Briefly state the recommendations and specify age range:<br>18-65 years:<br>min. 150 min.of moderate PA per week, opti. 300 min. of moderate PA per week<br>min. 75 min.of intensive PA per week, opti. 150 min. of intensive PA per week<br>Plus: strength exercises of large muscle groups and exercises shaping flexibility and mobility	

Older adults/seniors	Document/policy title and date: National recommendations for healthy activity, August 2018<br>Issuing body: Ministry of Sport and Tourism<br>Web link (English version not available): https://www.awf.katowice.pl/sites/default/files/uploads/Newsy/2018/raport_str_tyt.png<br>Briefly state the recommendations and specify age range: >65<br>min. 150 min.of moderate PA per week, opti. 300 min. of moderate PA per week<br>min. 75 min.of intensive PA per week, opti. 150 min. of intensive PA per week<br>Plus: exercises to improve balance and coordination, strength exercises of large muscle groups (if it is possible) and exercises shaping flexibility and mobility	
People with disabilities	Document/policy title and date:<br>Issuing body:<br>Web link (English version available?):<br>Briefly state the recommendations and specify age range:	X
Other (pregnat women):	Document/policy title and date: National recommendations for healthy activity, August 2018<br>Issuing body: Ministry of Sport and Tourism<br>Web link (English version not available): https://www.awf.katowice.pl/sites/default/files/uploads/Newsy/2018/raport_str_tyt.png<br>Briefly state the recommendations and specify age range:<br>4-7 times per week and more than 30 min. per day (optimal situation)	

17b. Does your country have any **national recommendations on reducing sedentary behaviour**?<br>**If recommendations exist for any of the target groups listed,** please provide details for each of the population subgroups (where applicable), including the issuing body, year of publication, title of the document, and provide a web link if available (please also specify whether the document is available in English).<br>**If no recommendations exist, please mark the "no" column for the respective target group.**		
		**No**
Early years (pre-school age)	Document/policy title and date:<br>Issuing body:<br>Web link (English version available?):<br>Briefly state the recommendations and specify age range:	X
Children and young people (school-age)	Document/policy title and date:<br>Issuing body:<br>Web link (English version available?):<br>Briefly state the recommendations and specify age range:	X
Adults	Document/policy title and date:<br>Issuing body:<br>Web link (English version available?):<br>Briefly state the recommendations and specify age range:	X
Older adults/seniors	Document/policy title and date:<br>Issuing body:<br>Web link (English version available?):	X

	Briefly state the recommendations and specify age range:	
People with disabilities	Document/policy title and date:<br>Issuing body:<br>Web link (English version available?):<br>Briefly state the recommendations and specify age range:	X
Other (please specify):	Document/policy title and date:<br>Issuing body:<br>Web link (English version available?):<br>Briefly state the recommendations and specify age range:	X

18. Does your country have any **national goals (or national targets) for population prevalence of physical activity? If yes,** please provide details of each target and the time frame. Please specify in which policy document(s) listed in Question 7 these goals are stated.<br>Please start with the most specific and measurable targets, followed by a listing or summary statement of any more general targets and goals for physical activity-related behaviours.
Examples:<br>"By 2010, 65% (2004: 60%) of the adult population will meet the international exercise standard."<br>"An increase in the number of children and youth who are physically active for at least 60 minutes per day."
The only example in Poland is the *Sport Development Profgramme 2020* which main objective is to create conditions for the development of sport and to promote health-enhancing physical activity. Although the objective is qualitative there are some quantitative indicators to be achieved in 2020 specified:<br>- Percentage of citizens who they never do sports relative to the average EU-28 (EU-28 = 100) should be not less than 100 (compering with 123,8 in 2013)<br>- Percentage of citizens who don't take any (other than sport) physical activity in relation to EU-28 average (EU28 = 100) shold be not less than 100 (compering with 116,6 in 2013)<br>- Percentage of citizens who do not declare participation in sports or recreation classes should be not less than 46 (compering with 54,1 in 2012).

19. Aside from any national goals and targets for population prevalence of physical activity or sedentary behaviour (already provided in previous questions), does your country have **any other goals and targets that directly or indirectly relate to physical activity promotion?**<br>For example, a goal for health professionals to screen more patients for physical activity, or a target to replace a percentage of car trips by cycling and walking.<br>**If so,** please give examples, indicate the time period for the desired change, if available, and state in which of the policy documents presented in Question 7 these appear.
No other examples

SECTION 6 Surveillance

20. Does your country have a **health surveillance or monitoring system** that includes measures of physical activity or sedentary behaviour?<br>**If yes,** please provide details according to age group (you may copy and paste as many response sections as needed). Please describe long-term general population surveys in: Question 20a (children and young people); Question 20b (adults) and Question 20c (older adults/seniors).<br>Please add more boxes if needed.
**20a. Children and young people**
**Name of survey 1**: Childhood Obesity Surveillance Initiative (COSI)

Methods used *(please tick as relevant)*	Survey		Interview		Objective measures*	Other method (please state)
	Paper	Online	Phone	Personal		
	X			X	X	

Please complete <u>either</u> row A or row B below, as relevant

A	Part of repeated surveillance system	Start year	Frequency	Latest year of data collection
		2016	1 year	2017
B	Single survey(s)	Year(s)	Meant as start of repeated surveillance system (yes/no or add comment)	

Name of survey 2: Health Behaviour in School Aged Children (HBSC)

Methods used *(please tick as relevant)*	Survey		Interview		Objective measures*	Other method (please state)
	Paper	Online	Phone	Personal		
	X			X	X	

Please complete <u>either</u> row A or row B below, as relevant

A	Part of repeated surveillance system	Start year	Frequency	Latest year of data collection
		2010	4 years	2018
B	Single survey(s)	Year(s)	Meant as start of repeated surveillance system (yes/no or add comment)	

* E.g. fitness, accelerometers

20b. Adults

Name of survey 1: European Health Interview Survey (EHIS)

Methods used *(please tick as relevant)*	Survey		Interview		Objective measures*	Other method (please state)
	Paper	Online	Phone	Personal		
	X			X	X	

Please complete <u>either</u> row A or row B below, as relevant				
A	Part of repeated surveillance system	Start year	Frequency	Latest year of data collection
		2009	5 years	2014
B	Single survey(s)	Year(s)	Meant as start of repeated surveillance system (yes/no or add comment)	

**Name of survey 2**:						
Methods used *(please tick as relevant))*	Survey		Interview		Objective measures*	Other method (please state)
	Paper	Online	Phone	Personal		

Please complete <u>either</u> row A or row B below, as relevant				
A	Part of repeated surveillance system	Start year	Frequency	Latest year of data collection
B	Single survey(s)	Year(s)	Meant as start of repeated surveillance system (yes/no or add comment)	

* E.g. fitness, accelerometers

**20c. Older adults**						
**Name of survey 1**: European Health Interview Survey (EHIS)						
Method used *(please tick as relevant)*	Survey		Interview		Objective measures*	Other method (please state)
	Paper	Online	Phone	Personal		
	X			X	X	

Please complete <u>either</u> row A or row B below, as relevant				
A	Part of repeated surveillance system	Start year	Frequency	Latest year of data collection
		2009	5 years	2014
B	Single survey(s)	Year(s)	Meant as start of repeated surveillance system (yes/no or add comment)	

**Name of survey 2**:						
Method used *(please tick as relevant)*	Survey		Interview		Objective measures*	Other method (please state)
	Paper	Online	Phone	Personal		

Please complete <u>either</u> row A or row B below, as relevant				
A	Part of repeated surveillance system	Start year	Frequency	Latest year of data collection
B	Single survey(s)	Year(s)	Meant as start of repeated surveillance system (yes/no or add comment)	

* E.g. fitness, accelerometers

21a. Have **data on the prevalence of physical activity or sedentary behaviour** or other related factors **influenced policy development** in your country?<br>For example, have surveillance data been used to define national goals and targets, or to assess progress towards achieving national goals and targets? **If yes,** please explain briefly and give examples.<br>**If no**, please briefly explain why. For example, is the frequency of data collection not in line with the timeline of formulated policy goals, or do the questions asked in the survey not provide information on the effectiveness of national policy implementation?
Yes. For example the new *Sport development programme 2020* was created on the basis of monitoring data and deep diagnosis of Polish society physical and sport activity.
21b. In your opinion, have **surveillance data helped to progress the national promotion of** physical activity in your country in any other ways?<br>For example, has a decline of physical activity levels helped to increase political attention, or created media attention?<br>**If yes,** please explain briefly, giving examples.
No, there is no evidence of such situation in Poland.

SECTION 7
Evaluation

22a. Has your country undertaken **evaluation of any of the national policies or action plans listed in Question 7**?<br>**If yes,** please state the title of the report, publisher and year published. Where available, please also provide a web link and indicate whether an English version/summary is available. Please provide brief details of the evaluation undertaken, what has been evaluated, the data collection methods, a summary of the results and how these were used (or not) to define new policy.
Title: *Sport development programme 2020*<br>Publisher and date: April, 2015<br>Web link (English version not available): https://www.msit.gov.pl/pl/sport/badania-i-analizy/dokumenty-strategiczne/7563,Dokumenty-strategiczne.html<br>Brief description of the approaches, results and their use:<br>The Sport Development Program 2020 is a medium-term strategic document specifying the objectives, piorities and directions of intervention in the area of sport. In 2016 the Implementation Document (ID) was prepared and adopted by the Minister of Sport and Tourism. DI is a catalog of tools (i.e. programs, projects and other undertakings) to achieve the objectives and directions of Sport Development Programme 2020 intervention. In accordance with the provisions of the programme, the monitoring system for its implementation is conducted in two ways. At the strategic level, monitoring of the implementation is based on horizontal implementation indicators that were recorded in the programmme. At the operational level, it is based on measures for the implementation of individual tools recorded in ID. The evaluation of the program for 2016 is available in details (see the web link in Polish).
22b. Has any **evaluation** of physical activity projects or interventions taken place **at the subnational level** (coordinated with or independent from the national level)?

**If yes**, please give a brief general overview of relevant processes. It is not expected to cover the whole range of activities but rather to give an indication and overview of the general approach taken at the subnational level.
No

23. Has any **economic evaluation** of interventions or **physical inactivity** (i.e. not reaching the minimum recommended level of physical activity) at national level been undertaken in your country?<br>**If yes,** please state the title of the report, publisher and year published. Where available, please also provide a web link and indicate whether an English version/summary is available. Please provide a brief description of the results of the assessment (about 50–100 words).
Title: *Sport development programme 2020*<br>Publisher and date: April, 2015<br>Web link (English version not available): https://www.msit.gov.pl/pl/sport/badania-i-analizy/dokumenty-strategiczne/7563,Dokumenty-strategiczne.html<br>Brief description of the approaches, results and their use:<br>The monitoring of implementation of the programme covers both the performance metrics for the implementation of individual measures as well as the amount of expenditure incurred for their implementation. The matrix with the amounts spent is available in details (see the web link in Polish).

SECTION 8
Funding and commitments

24a. Within each of the sectors listed, is **funding** specifically allocated or "ring-fenced" for the delivery of physical activity- related policy or action plans **at the national level**?
Please tick yes/no, and provide the amount (and currency), if known. Please also indicate whether this funding is recurrent; that is, provided on a regular basis (e.g. annually).

	**National**						
					**Recurrent**		
**Sector**	**Yes**	**Amount (PLN)**	**No**	**Don't know**	**Yes**	**No**	**Don't know**
Health	X	1 800 000			X		
Sport/recreation	X	265 400 000			X		
Education (higher education)	X	7 800 000			X		
Transport	X	64 500 000				X	
Environment				X			X
Urban design/planning				X			X
Other (Tourism)	X	1 000 000			X		
Other (Social affairs)	X	3 100 000			X		
Remarks or comments	Data for 201, amunt of money spent on realization of National Sport Strategy 2020						

24b. Within each of the sectors listed, is **funding** specifically allocated or "ring-fenced" for the delivery of physical activity- related policy or action plans **at the subnational level?**

Please tick yes/no, and provide the amount (and currency), if known. Please also indicate whether this funding is recurrent; that is, provided on a regular basis (e.g. annually).							
	**Sub-national**						
					**Recurrent**		
**Sector**	**Yes**	**Amount**	**No**	**Don't know**	**Yes**	**No**	**Don't know**
Health							
Sport /recreation							
Education							
Transport							
Environment							
Urban design / planning							
Other (please specify)							
Other (please specify)							
Remarks or comments	No information on the subnational level						

25. In your opinion, does evidence exist of **political commitment** to the national agenda to promote physical activity? This might include, for example: recognition of physical activity as an important policy topic; increased funding; inclusion of physical activity in official speeches; political discussions about physical activity promotion in parliament; visible engagement by politicians in HEPA-related events, or their personal participation in HEPA.<br>**If yes**, please describe, giving examples, and also comment on whether you think there is greater or less political commitment to physical activity promotion in your country than in the recent past.
No, there is no political commitment to promote physical activity in Poland

SECTION 9
Capacity-building through a national network

26. Does any professional **network or system exist in your country that links and/or supports professionals** interested or currently working in physical activity or related areas?<br>**If yes**, please describe, providing a web link and contact person, where available.
Yes, but mainly these are associations or informal networks, e.g.:<br>- The National Talent Base (a tool/databasis for gathering knowledge about the level of physical condition of children and youth as well as an instrument supporting the process of identifying sports talents)<br>- The Polish Active Mobility Union (PUMA) is oriented towards innovation, a nationwide organization of local governments - cities, poviats and communes interested in improving conditions and promoting all forms of active mobility.

SECTION 10
Experience of policy implementation, progress and remaining challenges

27a. What do you think are the areas of **greatest progress** in national HEPA promotion in your country in recent years?
1. Physical activity monitoring system in Poland
2. Sport infrastructure development - outdoor gyms programme (OSA)
3. Development of the habit of regular sport and physical activity in families
27b. What do you think have been the **biggest challenges** faced by your country in the commencement or continuation of national-level approaches to HEPA promotion in recent years?
1. Still the way how to measure the physical activity in Poland (e.g. among different age groups).
2. The lack of coordination of physical activity action between sectors and self-government.
3. The lack of broad HEPA promotion in mass media.

28. Based on your experience, please identify up to three **suggestions** you would offer to another country that is setting up a national HEPA policy.
1. The national HEPA focal point and HEPA activities coordinating organization should be launched.
2. The method of measurement of HEPA for individual age groups should be developed.
3. The national programme for HEPA promotion should be developed and implemented.

29. Please use this space to provide **any further details or comments** you were not able to provide in other sections of the tool.

SECTION 11
Summary of how the HEPA PAT was completed

It will be of interest to those who read this audit of HEPA policy to know how this review was undertaken and who was involved in the process. Please briefly outline the process used. This should include details of:

- who initiated the process
- who led the process
- who was involved
- how stakeholders were identified or selected
- the main steps of the consultation process.

In addition, please include a list of individuals and organizations that were contacted to provide feedback and indicate who responded. Please add/remove rows as needed.

**Lead of PAT completion process**
Name: Joanna Zukowska<br>Institution: Gdansk University of Technology<br>Contact details: Joanna.zukowska@pg.edu.pl
Name: Lech Michalski<br>Institution: Gdansk University of Technology<br>Contact details: michal@pg.gda.pl
**PAT completion team members**
Name: Lech Michalski<br>Institution: Gdansk University of Technology<br>Contact details: michal@pg.gda.pl
Name: Anna Gobis<br>Institution: Gdansk University of Technology<br>Contact details: anna.gobis@pg.gda.pl

**Overview of process and timelines**	
**Month/year**	**Main steps**
02/03 2019	JZ and LM wrote a first draft of the HEPA PAT based on phone consultancy with Ministry of Sport and Tourism (HEPA PAT focal point) as well as on additional desk research
04 2019	Prefill HEPA PAT using data from EU PA monitoring framework and used this to prefill the HEPA PAT
04 2019	JZ and LM continue the work based on the WHO Country Factsheets
05 2019	Workshop at the Ministry of Sport and Tourism (HEPA PAT focal point)
05-09 2019	JZ. LM and AG desk based research to complete prefill PAT
06-10 2019	Verification of HEPA PAT via survey
14 11 2019	Experts workshop in prefilled HEPA PAT
